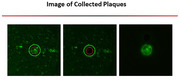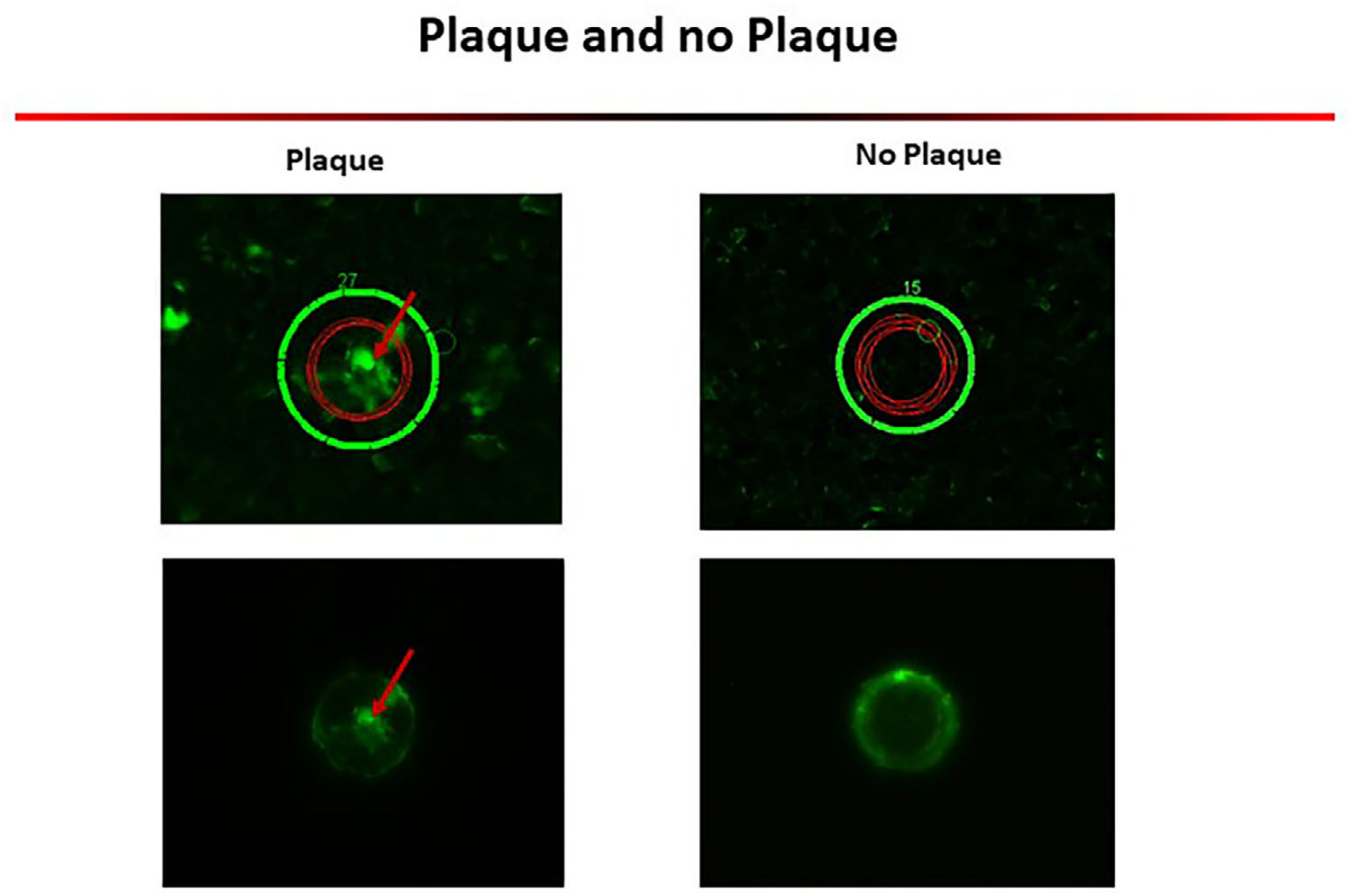# Spatial proteomic analysis of single amyloid plaques in brain tissue using laser capture microdissection and mass spectrometry

**DOI:** 10.1002/alz.095674

**Published:** 2025-01-09

**Authors:** yun Jiao

**Affiliations:** ^1^ St. Jude Childeren’s research hospital, Memphis, TN USA

## Abstract

**Background:**

Alzheimer’s disease (AD) is characterized by the accumulation of pathological amyloid protein deposits in the brain. Analyzing the proteomic composition amyloid plaques is essential for advancing biomedical research on Alzheimer’s disease. Laser capture microdissection (LCM) is a technique that enables precise isolation and collection of specific cells or structures from tissue samples. In this study, we developed a method utilizing LCM to capture single amyloid plaques from fresh frozen brain tissue for subsequent profiling using Data‐independent acquisition (DIA) proteomics.

**Methods:**

1: Tissue sections (10‐12 µm) were collected from fresh frozen mouse and human brain tissue and stored at ‐80°C.2: Fresh frozen tissue sections were fixed with 70% alcohol and stained for plaques using X‐34 dye with series dehydration. 3: One plaque and one non‐plaque areas from the same section were captured using LCM (approximately 50 µm diameter). 4: Perform proteomic analysis using a modified LC‐MS/MS method.

**Results:**

1: We developed an optimized method for isolating single amyloid plaques from fresh frozen brain tissue samples. 2: We established a proteomic platform for analyzing single plaque samples at a sub‐microgram level, representing one of the studies with the deepest coverage of single plaque proteome. This platform utilized liquid chromatography and tandem mass spectrometry (LC‐MS/MS) in conjunction with data‐independent acquisition (DIA) proteomic profiling. 3: Due to the low protein level in plaques (∼5ng/plaque), we use 0.2%DCA as a lysis buffer. This buffer can precipitate upon acidification, eliminating the need for a desalting step and minimizing sample loss. Finally, ∼20,000 peptides and ∼5,000 proteins have been identified from ∼5ng initial protein per sample. 4: Notably, we observed several key proteins, such as Abeta, Apoe, Mdk, and Ntn1, consistently appeared in the plaque areas. The enriched protein pathways and protein‐protein interaction modules were also reported.

**Conclusion:**

Our approach provides an optimized platform for conducting in‐depth profiling of LCM‐captured single amyloid plaque in Alzheimer’s disease. This method enables a comprehensive understanding of the proteomic composition of these plaques, contributing to the advancement of Alzheimer’s disease research.